# Multiconstrained gene clustering based on generalized projections

**DOI:** 10.1186/1471-2105-11-164

**Published:** 2010-03-31

**Authors:** Jia Zeng, Shanfeng Zhu, Alan Wee-Chung Liew, Hong Yan

**Affiliations:** 1 School of Computer Science and Technology, Soochow University, Suzhou 215006, China; 2 Department of Computer Science, Hong Kong Baptist University, Kowloon Tong, Hong Kong; 3 School of Computer Science and Technology, Fudan University, Shanghai 200433, China; 4 Shanghai Key Lab of Intelligent Information Processing, Fudan University, Shanghai 200433, China; 5 School of Information and Communication Technology, Griffith University, Gold Coast Campus, QLD 4222, Queensland, Australia; 6 Department of Electronic Engineering, City University of Hong Kong, Kowloon, Hong Kong; 7 School of Electronic and Information Engineering, University of Sydney, NSW 2006, Australia

## Abstract

**Background:**

Gene clustering for annotating gene functions is one of the fundamental issues in bioinformatics. The best clustering solution is often regularized by multiple constraints such as gene expressions, Gene Ontology (GO) annotations and gene network structures. How to integrate multiple pieces of constraints for an optimal clustering solution still remains an unsolved problem.

**Results:**

We propose a novel multiconstrained gene clustering (MGC) method within the generalized projection onto convex sets (POCS) framework used widely in image reconstruction. Each constraint is formulated as a corresponding set. The generalized projector iteratively projects the clustering solution onto these sets in order to find a consistent solution included in the intersection set that satisfies all constraints. Compared with previous MGC methods, POCS can integrate multiple constraints from different nature without distorting the original constraints. To evaluate the clustering solution, we also propose a new performance measure referred to as Gene Log Likelihood (GLL) that considers genes having more than one function and hence in more than one cluster. Comparative experimental results show that our POCS-based gene clustering method outperforms current state-of-the-art MGC methods.

**Conclusions:**

The POCS-based MGC method can successfully combine multiple constraints from different nature for gene clustering. Also, the proposed GLL is an effective performance measure for the soft clustering solutions.

## Background

Computational annotating gene functions is a fundamental issue in bioinformatics. Microarray gene expression data have been used widely to study the cell cycle system, genetic regulatory interactions, development at the molecular level, and genes that act in response to a certain infectious disease. To determine gene functions, a basic approach is gene clustering using gene expression data based on the assumption that genes with similar expression patterns should share similar functions in the process. Typical gene clustering methods include hierarchical clustering [1], the k-means algorithm [2], self-organizing maps [3], the fuzzy c-means algorithm [4], and hidden Markov models [5]. However, gene clustering regularized by only single constraint of gene expression is not enough to obtain biologically reliable clusters, because microarray data are often noisy, contain missing values, and have uncertain temporal dependencies in time-series data [6,7]. Therefore, other constraints besides gene expression data should be incorporated for the robust and reliable gene clustering.

Recent multiconstrained gene clustering (MGC) methods have attracted much more interests [8-13]. The basic idea is that multiple constraints such as Gene Ontology (GO) and metabolic network structures can prevent gene clustering from falling into the locally optimal solution space constrained by noisy gene expression data alone. One key problem is how to combine multiple pieces of constraints to find a consistent clustering solution. Current MGC methods adopt a linear combination strategy to integrate multiple constraints of the same nature into a single new constraint, so that standard clustering algorithms for single-constrained gene clustering problems can be used, e.g., hierarchical clustering [8], Gaussian mixture models [9], k-medoids [10], and iterative conditional modes (ICM) for Markov random fields [12]. More specifically, they build a distance matrix of gene expression data as the first constraint, and then build another distance matrix based on either metabolic pathway [8,12,14] or GO annotations [9,10] as the second constraint. These two constraints of distance matrices are added linearly to form the new distance matrix for gene clustering. This linear combination strategy has also been used to incorporate different constraints in document clustering [15,16]. Despite good clustering performance, there are two major problems yet to be solved. The first is that these MGC methods can only combine constraints of the same nature, i.e., all constraints have to be represented as distance matrices. If one constraint is a similarity matrix, we need to transform it into a distance matrix so that we can add it up to other distance matrices. Such transformation may distort the original constraint with information loss. Even if we have two distance matrices, the distance values may be in different scales and cannot be added directly. The second problem lies in the linear combination of the constraint matrices. In most cases, the desired combined constraint does not necessarily have a simple linear relationship with all other original constraints. In addition, the weights for the linear combination often need a reasonable justification in practice. Another MGC strategy is the GO-guided fuzzy c-means (FCM) algorithm [13], which uses GO annotations to initialize and update the cluster probability of each gene.

To overcome above problems, we propose a novel MGC method within the generalized projection framework, which is a generalization of the projection onto convex sets (POCS) technique, which has found many applications in image reconstruction [17] and microarray missing value imputation [18]. Theoretically, POCS provides a flexible framework to integrate multiple pieces of constraints for an optimal solution. It first transforms each constraint into a corresponding convex set, and then uses an iteratively convergent procedure to find a solution in the intersection of all sets. POCS can integrate constraints from different nature such as different similarity matrices. Indeed, it often handles different constraints in frequency and spatial domains in image reconstruction problems. Another advantage is that the original constraints remain intact. The clustering result is projected onto the solution set that satisfies each constraint iteratively and the final result may lie in the intersection set that satisfies a nonlinear combination of the original constraints. Without loss of generality, in this paper we consider two major types of constraints: the gene expression similarity [8] and the GO-based semantic similarity [19]. POCS produces a regularized clustering result that may be more reliable than those solely dependent on either the gene expression similarity or the GO semantic similarity due to the fact that expression data are often short and noisy, while GO terms may be inaccurate and mis-annotated. Because in most cases the solution set is nonconvex, we adopt the generalized projections similar to the POCS procedure. To minimizes the distance between the candidate solution and the constraint set, we design the generalized projector based on a method similar to the relaxation labeling (RL) algorithm [20,21], which has been used for the approximate inference for Markov random fields [22,23]

Usually genes have multiple functions and can be assigned into more than one group. Traditional gene clustering algorithms often use a hard clustering strategy that assigns genes into only one group. Recent MGC methods relax this limitation and allows genes to be assigned into several groups [9,10,13]. To take this situation into account, we use a soft clustering strategy in which genes are assigned to all clusters with different probabilities. Based on soft clustering results, we propose a new performance measure "gene log likelihood" (GLL) to measure the distance between the predicted clustering result and the reference clusters. This measure has also been widely applied to evaluating word clustering performance in topic modeling problems [24]. To confirm the effectiveness, we evaluate the POCS-based MGC method on the yeast gene expression dataset, and compare the clustering results with recent MGC methods such as k-medoids [10], ICM [12] and FCM [13]. Experimental results demonstrate that the POCS-based MGC can enhance the overall clustering performance by a large margin.

This paper is organized as follows. In the next section we propose the POCS-based MGC method and the RL-based generalized projector to minimize the distance between clustering solution to the corresponding constrained solution set. To account for genes in multiple clusters, we also propose GLL for calculating the distance between the predicted soft clustering results and the reference gene clusters. The result section shows comparative experimental results on different yeast expression datasets. The POCS-based MGC algorithm always converges to the optimal solution in practice. Finally, we draw conclusions and envision future work.

## Methods

Gene clustering is a labeling problem, in which a set of cluster labels are assigned to genes for annotating gene functions. Given *I *genes and *K *clusters, the soft clustering solution is a matrix **X **= (*x*_*ik*_), 1 ≤ *i *≤ *I*, 1 ≤ *k *≤ *K*, where *x*_*ik*_∈ [0, 1] and Σ_*k *_*x*_*ik *_= 1. The element *x*_*ik *_is the probability that the *i*th gene is associated with the *k*th cluster label. For each gene we use a probability vector **x**_*i *_= (*x*_*i*1_, . . ., *x*_*ik*_, . . ., *x*_*iK*_) to represent its cluster labeling configuration. From this perspective, the clustering solution **X **is the cluster labeling configuration of *I *genes over *K *clusters. We may also use the winner-take-all strategy to figure out the hard clustering solution X*, in which the *i*th gene belongs to only one cluster with the highest probability, i.e., k* = arg max_*k *_*x*_*ik *_and *x*_ik* _= 1.

### Gene expression constraint

Based on microarray gene expression profiles, we can build the first constraint using the similarity matrix for gene clustering. The metric can be the Pearson's correlation coefficient and Euclidean distance [8-10], or the more complex type-2 fuzzy hidden Markov model-based sequence similarity [25]. Because the Pearson's correlation coefficient is suitable for time-series gene expression data [26], we adopt it for calculating the similarity between two genes' log-ratio transformed profiles [8], i.e., the logarithm of the ratio between each sample point in the profile and a control measurement. More specifically, given two genes' transformed profiles *g*_*i*_(*m*) and *g*_*i*'_(*m*) in length *M*, the correlation coefficient *v*_*ii*' _is

where *μ*_*i *_and *σ*_*i *_denote mean and standard deviation of the transformed profile of the *i*th gene respectively. The correlation coefficient value  ∈ [-1, 1], where the higher value corresponds to the higher similarity between two genes' profiles. Here we consider the anti-correlated gens as most dissimilar because the correlated genes often involve in similar reaction steps and share similar functions. Therefore, the Pearson's correlation coefficient matrix  constrains the first clustering solution set *C*_1 _= {**X**_*e*_}, which contains many locally optimal clustering solutions satisfying .

### GO constraint

As an important source of biological knowledge, the Gene Ontology (GO) provides a consistent description of genes and gene products by a controlled and structured vocabulary, which includes three major categories: biological process (BP), molecular function (MF), and cellular component (CC). The GO terms are organized in the form of a directed acyclic graph (DAG) with two major semantic relations such as "is-a" and "part-of", where "A *is-a *B" means A is a subclass of B, and "C *part-of *D" means C is always part of D. Generally, simply identifying the shared GO annotations of gene products for their functional relationship has the following limitations. First, two quite different GO annotations can be closely related through their common ancestors in the DAG so as to have a higher semantic similarity. Second, the shared GO terms may be too general to describe the functional association of annotated gene products. Recently, the GO-based semantic similarity measures have been applied to searching semantically similar proteins [27], clustering gene expression data and assessing cluster validity [19,28,29], developing new human regulatory pathway modeling tools [30], validating protein interaction data [31], validating functional annotation of expression-based clusters [32], and enabling the identification of functionally related gene products independent of homology [33].

The GO-based semantic similarity measures assume that the more information two GO terms share, the more similar they are. In this paper we adopt a recent GO-based semantic measure proposed by Wang et al. [19], in which the similarity between two GO terms *S*_*GO*_(*c*_*m*_, *c*_*n*_) is calculated according to the graph structural information encoded in the GO. This semantic measure between annotated GO terms for genes has been demonstrated to be better than the classic Resnik's measure in clustering gene products. If *c *is a GO term,  is the set of GO terms including term *c *and all its ancestors, and *E*_*c *_is the set of edges connecting all terms in , the S-value of any term *t *in the graph DAG_*c *_= (*c*, , *E*_*c*_) related to the term *c*, *S*_*c*_(*t*), is defined as,

where *w*_*e *_is the semantic contribution factor for edge *e *∈ *E*_*c *_linking the term *t *with its child term *t*'. Here we use *w*_*e *_= 0.8 for "is-a" relation and *w*_*e *_= 0.6 for "part-of" relation as suggested in [19]. After obtaining all S-values for all terms in the DAG_*c*_, the semantic value of the term *c*, *SV *(*c*), is

Given two GO terms *c*_1 _and *c*_2 _as well as their graphs  and , the semantic similarity *S*_*GO*_(*c*_1_, *c*_2_) is

where  (*t*) is the S-value of GO term *t *related to term *c*_1_, and  (*t*) is the S-value of GO term *t *related to term *c*_2_. One gene may be annotated by many GO terms. Given two genes annotated by several GO terms, *GO*_*i *_= {*c*_*i*1_, . . ., *c*_*im*_, . . ., *c*_*iM*_} and *GO*_*i*' _= {*c*_*i*'1_, . . ., *c*_*i*'*n*_, . . ., *c*_*i*'*N*_}, the functional similarity between genes,

Note that the functional similarity  between two GO term sets *GO*_*i *_and *G*_*Oi*' _considers the hierarchical structure of GO terms c based on the S-value. Because the GO contains three main vocabularies, BP, MF and CC, the GO similarity value between genes can be calculated in a joint manner as

where BPsim, MFsim and CCsim denote the similarity values  of the corresponding GO terms within the same type. The similarity value  ∈ [0, 1], where the higher value corresponds to the higher similarity. As a result, the GO-based semantic similarity  constrains the second clustering solution set *C*_2 _= {**X**_*g*_}, which contains many locally optimal clustering solutions satisfying .

### Generalized projections

Although the gene expression and GO-based semantic similarity may achieve a clustering solution with a high correlation, there is still a large amount of complementary information between their final clustering results [34]. Both gene expression and GO constrained solution sets *C*_1 _= {**X**_*e*_} and *C*_2 _= {**X**_*g*_} may not contain a single globally optimal solution, and even they contain such a solution, we are unlikely able to find it since the optimization procedures are highly nonlinear. So, we consider *C*_1 _and *C*_2 _as sets of all locally optimal solutions under different constraints. When both constraints are satisfied, we eliminate many unreasonable locally optimal solutions and obtain an improved clustering performance. Our objective is to find the biologically consistent clustering solution **X**^†^∈ *C*_1 _∩ *C*_2 _using the POCS procedure [17]. Note that direct adding two constraints  and  based on the weight *w *∈ [0, 1], i.e., , to produce the new constraint for gene clustering is not suitable because the constraints are from different nature. In contrast, the POCS framework decomposes the optimization procedure into different projections and solves the problem efficiently.

**input**: **X**_0_, *P*_*n*_, *w*_*n*_, 1 ≤ *n *≤ *N*, *M*.

**output**: **X**_*M*_.

begin

   **for ***m *← 1 **to ***M ***do**

     ;

     // *P*_*n*_*X*_*m*-1 _is described in Algorithm 2.

   **end**

end

**Algorithm 1: **The simultaneous projection.

Within the POCS framework [17], each constraint on the solution is formulated as a corresponding closed convex set, *C*_*n*_, 1 ≤ *n ≤ N*, in the Hilbert space **H**. The optimal solution **X**^† ^is included in the intersection set *C*_0 _of all convex sets *C*_*n*_,(1)

**Figure 1:**
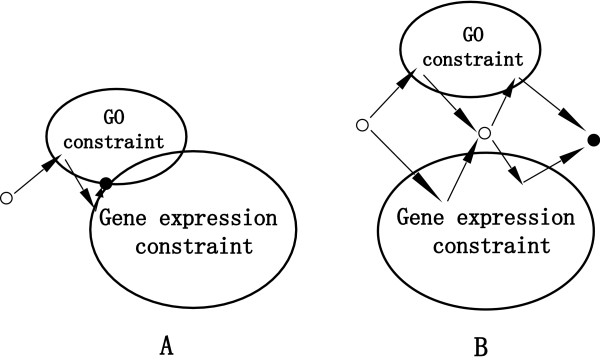
**(A) The consistent problem in Eq**. (2), where the intersection set *C*_0 _is nonempty. The circle is the initial solution. The thick black point is the consistent solution in the intersection of two sets for gene expression and GO constraints, respectively. POCS ensures that the initial solution will converge to the consistent solution after enough projections represented by the arrows. (B) The inconsistent problem in Eq. (4), where the intersection set *C*_0 _is empty. After enough simultaneous projections represented by the arrows, the thick black dot is the approximate solution such that a weighted set distance from gene expression and GO constraints is minimized.

If *C*_0 _is nonempty in Figure 1A, the successive projections onto the convex sets,(2)

will converge to a consistent solution in *C*_0 _for any random initial value **X**_0_, where **X**_*m*_, 1 ≤ *m *≤ *M *is the solution at the mth iteration. Eq. (2) shows that the current solution **X**_*m*-1 _is projected to each set or constraint *C*_*n*_, 1 ≤ *n *≤ *N *through the projector *P*_*n *_successively in order to find the next better solution **X**_*m *_until it converges to the consistent solution **X**^† ^in the intersection of all sets. Figure 1A shows the projection process for the consistent problem in Eq. (2), where the thick black dot represents a consistent solution in the intersection of two sets *C*_1 _and *C*_2 _for the gene expression and GO constraints, respectively. The generalized projector *P*_*n *_transforms **X**_*m*-1 _into a solution  within the set *C*_*n *_that minimizes the distance between **X**_*m*-1 _and ,(3)

where ||· || denotes the norm in the Hilbert space **H**. Indeed, Eq. (3) indicates that we need to transforms the current clustering solution **X**_*m*-1 _into a more suitable clustering solution  based on the similarity or distance matrix  for the set *C*_*n*_. If *C*_0 _is empty in Figure 1B, the POCS algorithm uses simultaneous projections,(4)

where *w*_*n *_is the weight on the projections satisfying  and *w*_*n *_≥ 0 for all *n*. The simultaneous projections converge weakly to a solution such that a weighted set distance function is minimized. Note that the simultaneous projections only linearly combine the solutions projected onto all constraint sets, which is more reasonable than the strategy that linearly combines constraints and then finds a solution under the new constraint. Figure 1B shows the simultaneous projections for the inconsistent problem in Eq. (4), where the thick black dot is an approximately best solution minimizing the weighted set distance from gene expression constraint *C*_1 _and GO constraint *C*_2_, respectively.

In practice, both *C*_1 _and *C*_2 _are often nonconvex. A set is convex if and only if *λ***X**_*a *_+ (1 - *λ*)**X**_*b *_is in the set when **X**_*a *_and **X**_*b *_are in the set for 0 ≤ *λ *≤ 1. The constraint sets contain many locally optimal clustering "solutions" and the interpolation of the solutions, i.e., the weighted sum *λ***X**_*a *_+ (1 -*λ*)**X**_*b*_, has no mathematical meaning. Thus, we cannot use the classic POCS procedure (2). Nevertheless, we can still use the generalized projections (3) to solve the problem within the POCS framework [[17], Chapter 5], which do not require the sets be convex. In practice it is difficult to minimize the distance functions (3) under both constraints at the same time, so we do it iteratively based on generalized projections. The generalized projector iteratively minimizes the distance function (3), and will terminate if the distance in the next step cannot decrease. From the regularization point of view, the solution is regularized under different constraints simultaneously, and the final solution is a linear combination of each regularized solution in Eq. (4). The simultaneous projection weights *w*_*n *_can be fixed empirically according to prior knowledge. To summarize, Algorithm 1 shows the simultaneous projection algorithm.

**input**: , 1 ≤ *i*, *i*' ≤ *I*, 1 ≤ *k *≤ *K*, *J*.

**output**: , 1 ≤ *i *≤ *I*, 1 ≤ *k *≤ *K*.

begin

   **for ***j *← 1 **to ***J ***do**

     **for ***i *← 1 **to ***I ***do**

       **for ***k *← 1 **to ***K ***do**

         ;

         ;

       **end**

     **end**

   **end**

end

**Algorithm 2: **The relaxation labeling projector.

Now we design the generalized projector based on the iterative RL algorithm [20,21,23], which can find the soft cluster label for each gene under a certain constraint. Given the clustering solution **X **and the constraint , minimizing (3) is equivalent to maximizing the corresponding gain function,(5)

where *i' *∈ ∂_*i *_is a set of neighbors of the *i*th gene, and the term exp() increases with the similarity between two genes according to the constraint . The neighborhood system ∂_*i *_is defined as the ten nearest genes *i' *with top similarity values  . The term  encourages that if the genes have a high similarity value  they also have a high similarity value in soft cluster labeling configurations. The RL algorithm iteratively updates the initial **X**^1 ^by the gradient  of the gain function (5) until *j *reaches the fixed maximum number *J *as shown in Algorithm 2. The value of *J *is determined experimentally to ensure that the gain function is maximized. That is, after *J *iterations, the RL algorithm converges to the local maximum of the gain function in terms of **X**^*J*^. In the meanwhile, the distance function (3) is also minimized by **X**^*J*^, where **X**^*J *^is equivalent to  in (3). Algorithm 2 shows the projection of **X**^1 ^satisfying one constraint . Note that *J *is the number of iterations of the RL-based projector in Algorithm 2, while *M *is the number of iterations in the simultaneous projection in Algorithm 1. The RL-based projector is a fast algorithm and practically *J *= 5 is enough.

### Gene log likelihood

If we have a reference gene clustering solution **Y**, we can calculate the distance between the predicted clustering solution **X **and the standard reference **Y **for the performance evaluation. The reference clustering solution is a matrix, **Y **= (*y*_*iw*_), 1 ≤ *i *≤ *I*, 1 ≤ *w *≤ *W*, where *y*_*iw *_= 1 denotes that the *i*th gene belongs to the *w*th cluster. The number of reference clusters W may not equal to the predicted number of clusters K in most cases. Because a gene may belong to multiple clusters due to multiple functions, the vector **y**_*i *_= (*y*_*i*1_, . . ., *y*_*iw*_, . . ., *y*_*iW*_) may contain multiple ones for the *i*th gene.

Based on the hard clustering solution X*, we may quantify the distance between X* and **Y **by normalized mutual information (NMI), which has been widely used in a lot of applications to measure the performance of clustering methods [12,19]. In information theory, the mutual information is defined as a quantity to measure the amount of information shared between two random variables. If one set of clusters is more consistent with the other set of clusters, the mutual information between two sets of cluster labels becomes larger. Generally, the mutual information is normalized because the range of the mutual information measures depends on the size of given sets of clusters. NMI is calculated as

where *I *is the number of genes, *n*_*w *_is the number of genes in the *w*th reference cluster, *n*_*k *_is the number of genes in the *k*th reference cluster, and *n*_*wk *_is the number of genes in both *w*th reference cluster and *k*th predicted cluster. If two sets of clusters are identical, NMI between them reaches the maximum value of one.

However, NMI cannot be used if one gene may be in multiple clusters. So, we propose a new performance measure referred to as gene log likelihood (GLL) log *P*(**Y**|**X**) for gene clustering, which measures the likelihood in predicting a single gene in the reference cluster **Y **based on **X**. GLL has a simple meaning that the *i*th gene in the *w*th reference cluster **Y **is predicted with a likelihood proportional to the product of the likelihood that the *w*th cluster is generated by the *k*th cluster and the likelihood that the *i*th gene is generated by the *k*th cluster in **X**. Higher values are better, indicating the obtained clustering solution **X **has a higher likelihood to generate the reference gene clusters **Y**. Specifically we calculate GLL as follows,(6)

where **x**_*i *_= (*x*_*i*1_, . . ., *x*_*ik*_, . . ., *x*_*iK*_) is the probability distribution over *K *clusters of the *i*th gene, *i *∈ *w *denotes the set of all genes in the *w*th reference cluster with *y*_*iw *_= 1, and **p**_*w *_= (*p*_*w*1_, . . ., *p*_*wk*_, . . ., *p*_*wK*_) is the probability distribution of the wth reference cluster over *K *predicted clusters. Empirically, this probability *p*_*wk *_can be estimated by(7)(8)

where we assume that the genes are conditionally independent in the generative process. Indeed, this is a standard performance measure for word clustering in the text mining [24], which indicates the empirical likelihood in predicting a single word in a document.

## Results and Discussion

### Datasets

To calculate the gene expression constraint, we select four microarray time-series datasets [35], monitoring genome-wide mRNA levels for 6178 yeast *Saccharomyces cerevisiae *open reading frames simultaneously using several different methods of synchronization including four datasets: alpha, cdc15, cdc28 and elu datasets. Also we add the Hughes dataset [36] widely used in gene clustering [9,10], because it contains 300 time points while a small number of missing values. The missing values in the microarray data are interpolated by the POCS-based reconstruction method [18], which uses multiple constraints such as synchronization loss. To calculate the GO constraint, the GO (version 20080225) and annotation (version 1.1384) databases of yeast are downloaded from the GO official website. The yeast annotation file includes 6345 gene products annotated with 77152 GO terms.

To evaluate MGC methods for gene clustering, we generate two different sets of reference gene clusters with true cluster labels from KEGG [37] and SGD (Saccharomyces Genome Database) http://www.yeastgenome.org/ referred to as KEGG clusters [12] and SGD clusters [19], respectively. The KEGG pathway maps are generally classified into six major categories including metabolism. We use ten subcategories under the metabolism category as KEGG clusters, which includes a total of 531 genes. Note that a gene can be in more than one cluster. Table 1 lists the KEGG clusters and the number of genes in the corresponding cluster. We also use the gene annotation and classification information in yeast biochemical pathways as SGD clusters. There are 142 pathways involved with 835 genes, among which only 26 pathways contain more than 10 genes, where a gene can be in more than one pathway. Table 2 summarizes the list of pathway clusters and the number of genes in the corresponding cluster. The reason why we use two different sets of reference clusters lie in the fact that gene clusters are variable depending on the different partitioning criteria. If the predicted clusters by the POCS-based method are close to both reference clusters, we may make a safe conclusion that this method is robust to annotate gene functions under different conditions.

**Table 1:** 10 reference gene clusters from KEGG

Cluster name	Number of Genes
Amino acid metabolism	197
Carbohydrate metabolism	189
Metabolism of cofactors vitamins	47
Energy metabolism	66
Glycan biosynthesis and metabolism	21
Lipid metabolism	74
Nucleotide metabolism	103
Metabolism of other amino acids	50
Metabolism of secondary metabolites	18
Xenobiotics biodegradation and metabolism	19

**Table 2:** 26 reference gene clusters from yeast biochemical pathways

Cluster name	Number of genes
TCA cycle, aerobic respiration	24
de novo biosynthesis of purine nucleotides	32
de novo biosynthesis of pyrimidine deoxyribonucleotides	15
de novo biosynthesis of pyrimidine ribonucleotides	12
ergosterol biosynthesis	15
fatty acid biosynthesis, initial steps	12
fatty acid oxidation pathway	11
folate biosynthesis	24
folate interconversions	17
folate polyglutamylation	13
folate transformations	16
gluconeogenesis	17
glycolysis	14
glyoxylate cycle	12
inositol phosphate biosynthesis	14
isoleucine degradation	13
lipid-linked oligosaccharide biosynthesis	15
pantothenate and coenzyme A biosynthesis	11
phenylalanine degradation	12
phosphatidylinositol phosphate biosynthesis	21
protein modifications	12
salvage pathways of adenine, hypoxanthine, and their nucleosides	11
sphingolipid metabolism	23
superpathway of glucose fermentation	14
tryptophan degradation	12
valine degradation	11

### Comparative results

The POCS-based MGC method requires two key parameters, the number of simultaneous projections M and the weight on projections *w*_*n*_, in Algorithm 1. Because we have two constraints, the weight for the GO-based constraint is *w*, and thus the weight for the gene expression constraint is 1 - *w*. Through experiments on the alpha dataset, we can determine proper *M *and *w *for desirable gene clustering performance. The parameters *M *and *w *are adjusted so that we can obtain the desirable result within the POCS framework. It is possible that another iterative method can estimate the parameters better. However, in many cases, such a better-performing method is a supervised learning procedure using reference gene clusters, and can be incorporated into the POCS procedure to achieve an even better performance or robustness. That is, POCS is useful for combining information from different sources if we can formulate corresponding constraint sets and projections.

**Figure 2:**
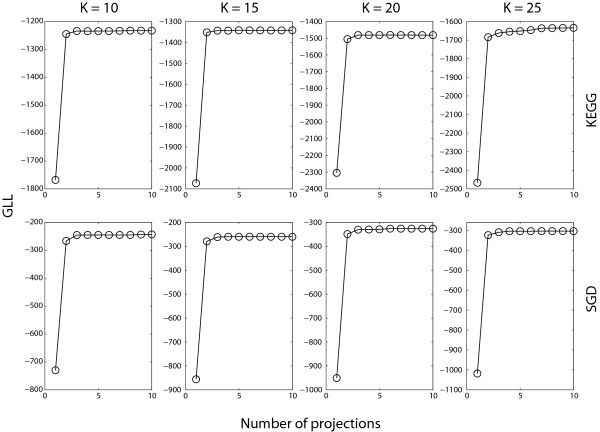
**GLL of the alpha dataset on the KEGG and SGD when 10 projections are used**.

To determine *M*, we randomly initialize the clustering solution, and the weight *w *= 0.5. Figure 2 shows the GLL values on the KEGG and SGD reference clusters when 10 projections are used. From different number of clusters *K *= 10, 15, 20, 25, we see that all GLL values do not increase significantly after two or three projections. So, we believe that *M *= 3 is enough to produce desirable clustering results in this task. From this experiment, we also see that Algorithm 1 converges quickly after a few projections. Then, we fix *M *= 3 and tune the weight *w *∈ [0, 1]. By using *M *= 3 projections in practice, POCS does not increase the computational cost very much, which makes this algorithm very attractive in combining more constraints for gene clustering.

**Figure 3:**
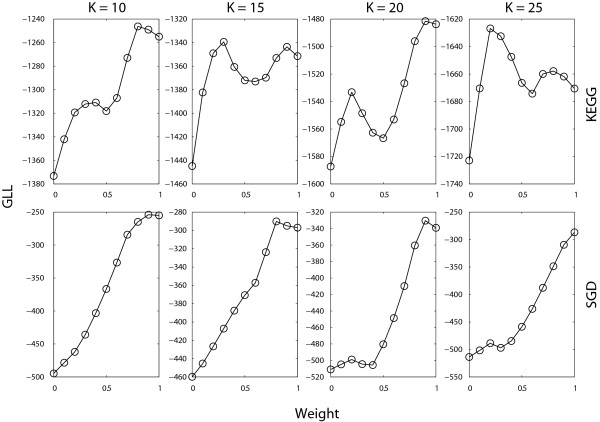
**GLL of the alpha dataset on the KEGG and SGD when different weights *w *are used**.

Figure 3 shows the GLL values on the KEGG and SGD reference clusters by increasing the weight at the step 0.1. We observe that the performance highly depends on different projection weights. If we use KEGG reference clusters, we find that weight *w *= 0.7 can produce higher GLL value on average. The gene expression constraint alone *w *= 0 does not ensure the best clustering result, while the GO constraint alone *w *= 1 does not ensure the best clustering result either. We see that the GO constraint can produce more reliable clustering result than the gene expression constraint, because the GO annotation is based on prior knowledge of biologists more reliable than gene expression data. Furthermore, we often assume that anti-correlated genes are not within the same cluster, but in some cases this assumption is not true. However, when the weight w increases, the final performance does not always increase and *w *= 0.5 produces a local minimum of the GLL value. After that, the GLL value continue to increase to the next local maximum of the GLL value. The SGD reference cluster reconfirms that the GO-based constraint is more reliable. The best clustering performance occurs often when *w *= 0.9 on average. Therefore, we adopt the weight *w *= 0.8 for the simultaneous projection in all our experiments.

As far as Figure 3 is concerned, one major reason why GO information is more reliable for clustering is that the reference gene clusters from KEGG and SGD (Tables 1 and 2) are partly correlated with GO annotations. Therefore, we need to delete a certain fraction of GO annotations when perform clustering, and use only the gene expression constraint to predict the new gene functions compared with reference gene clusters. In this paper, we adopt the cross-validation procedure [10] to validate the POCS-based MGC method. More specifically, we perform a five-fold cross-validation by deleting 20% GO constraints from the datasets in turn. We shall examine whether the POCS-based MGC clustering method can predict the functions for those 20% genes without GO constraints as compared to reference KEGG and SGD gene clusters. We repot the average prediction performance for the five-fold cross-validation.

After we fix *M *= 3 and *w *= 0.8, we compare our POCS-based MGC method with three state-of-the-art MGC methods: k-medoids [10], ICM [12] and FCM [13]. Both k-medoids and ICM first linearly combine two constraints  and , and then use the ICM and k-medoids algorithms to partition the genes into different clusters. We empirically determine the linear combination weight of the GO constraint *w *= 0.9 for k-medoids, which can produce the desirable clustering results in terms of GLL on average. For the ICM algorithm [12], we choose the best recommended parameter *w *= 0.2, which is biased toward the gene expression constraint. On the other hand, FCM uses GO annotations to initialize **X**_0_, and uses both initial **X**_0 _and gene expression values to update **X**_0 _until it converges to a new clustering solution **X**_*M *_. We use the best suggested weight *w *= 0.8 for FCM [13], which is biased toward the GO constraint for soft clustering.

Tables 3, 4, 5 and 6 show the average clustering performance and standard deviation in terms of GLL and NMI based on soft clustering solution **X **and the hard clustering solution X*, respectively. We see that the POCS produces the highest GLL value among all MGC methods, which means that its soft clustering solution is the most likely to generate both KEGG and SGD reference clusters. The k-medoids algorithm performs the worst, partly because it is easy to fall into the local optimal clustering solution. ICM uses an iterative procedure to find a better clustering solution by the combined constraint, but it is biased to the unreliable gene expression constraint. FCM performs slightly better than ICM partly because it is biased to the more reliable GO constraint. Compared with FCM, POCS significantly increases the GLL value around 15% on both KEGG and SGD reference clusters. Another observation is that the Hughes dataset has the highest GLL value, partly because it contains much longer gene expression profiles than alpha, cdc15, cdc28 and elu datasets. The longer gene expression profiles are more reliable for gene clustering. The NMI values are consistent with the GLL values, where if the soft clustering solution has a higher GLL value the corresponding hard clustering solution by the winner-take-all strategy also has a higher NMI value. Thus, the performance measure GLL can best account for this soft clustering solution, where the higher GLL value corresponds to better soft clustering solution. However, we observe that the GLL value varies much more than the NMI value, mainly because the soft clustering solution space is larger than that of the hard clustering. In some cases, the difference of NMI values between POCS and FCM is not significant. Thus, we need to examine the statistical significance in the difference of NMI values between POCS and FCM. Table 7 shows the p-values of pairwise *t*-test [38] over all five microarray datasets, which indicates that the NMI value of POCS is higher than the corresponding FCM results with a statistical significance of more than 99% for all datasets.

**Table 3:** Five-fold cross-validation of the GLL values on KEGG clusters

Datasets	POCS	**k-medoids **[10]	**ICM **[12]	**FCM **[13]
(a) *K *= 10				
alpha	-198 ± 8	-354 ± 9	-253 ± 14	-238 ± 14
cdc15	-194 ± 7	-372 ± 22	-220 ± 8	-212 ± 12
cdc28	-200 ± 9	-340 ± 20	-265 ± 8	-244 ± 10
elu	-199 ± 6	-355 ± 14	-253 ± 10	-228 ± 10
Hughes	-191 ± 4	-329 ± 17	-212 ± 5	-196 ± 9

(b) *K *= 15				
alpha	-184 ± 6	-415 ± 32	-282 ± 12	-262 ± 16
cdc15	-182 ± 4	-413 ± 28	-278 ± 10	-255 ± 15
cdc28	-189 ± 9	-424 ± 18	-294 ± 9	-271 ± 11
elu	-187 ± 9	-410 ± 35	-297 ± 11	-291 ± 13
Hughes	-180 ± 8	-401 ± 9	-262 ± 6	-234 ± 10

(c) *K *= 20				
alpha	-243 ± 10	-461 ± 27	-288 ± 11	-254 ± 22
cdc15	-225 ± 10	-460 ± 26	-271 ± 8	-246 ± 14
cdc28	-248 ± 8	-478 ± 33	-301 ± 9	-270 ± 10
elu	-259 ± 10	-476 ± 35	-304 ± 7	-286 ± 13
Hughes	-222 ± 6	-455 ± 34	-276 ± 9	-239 ± 13

(d) *K *= 25				
alpha	-304 ± 13	-494 ± 26	-363 ± 18	-328 ± 13
cdc15	-302 ± 6	-491 ± 41	-369 ± 12	-331 ± 17
cdc28	-298 ± 8	-444 ± 37	-363 ± 14	-326 ± 17
elu	-321 ± 7	-535 ± 23	-378 ± 9	-342 ± 13
Hughes	-284 ± 7	-478 ± 19	-351 ± 11	-319 ± 11

**Table 4:** Five-fold cross-validation of the NMI values on KEGG clusters

Datasets	POCS	**k-medoids **[10]	**ICM **[12]	**FCM **[13]
(a) *K *= 10				
alpha	0.287 ± 0.008	0.234 ± 0.007	0.251 ± 0.005	0.265 ± 0.005
cdc15	0.282 ± 0.003	0.222 ± 0.009	0.259 ± 0.002	0.268 ± 0.009
cdc28	0.267 ± 0.009	0.226 ± 0.005	0.209 ± 0.003	0.236 ± 0.003
elu	0.263 ± 0.006	0.219 ± 0.004	0.215 ± 0.001	0.240 ± 0.006
Hughes	0.289 ± 0.006	0.238 ± 0.007	0.254 ± 0.007	0.271 ± 0.005

(b) *K *= 15				
alpha	0.310 ± 0.009	0.255 ± 0.010	0.260 ± 0.010	0.283 ± 0.007
cdc15	0.305 ± 0.004	0.266 ± 0.004	0.278 ± 0.012	0.281 ± 0.001
cdc28	0.301 ± 0.001	0.266 ± 0.009	0.263 ± 0.008	0.279 ± 0.001
elu	0.292 ± 0.007	0.234 ± 0.002	0.244 ± 0.006	0.264 ± 0.009
Hughes	0.322 ± 0.003	0.286 ± 0.001	0.285 ± 0.007	0.303 ± 0.008

(c) *K *= 20				
alpha	0.382 ± 0.005	0.331 ± 0.001	0.335 ± 0.007	0.361 ± 0.004
cdc15	0.384 ± 0.002	0.339 ± 0.004	0.341 ± 0.003	0.367 ± 0.004
cdc28	0.361 ± 0.003	0.322 ± 0.001	0.336 ± 0.009	0.350 ± 0.007
elu	0.354 ± 0.007	0.311 ± 0.002	0.325 ± 0.003	0.342 ± 0.003
Hughes	0.396 ± 0.009	0.326 ± 0.003	0.356 ± 0.005	0.376 ± 0.009

(d) *K *= 25				
alpha	0.348 ± 0.008	0.307 ± 0.008	0.321 ± 0.008	0.339 ± 0.007
cdc15	0.353 ± 0.005	0.312 ± 0.002	0.309 ± 0.009	0.330 ± 0.009
cdc28	0.351 ± 0.003	0.316 ± 0.009	0.302 ± 0.009	0.336 ± 0.006
elu	0.338 ± 0.007	0.290 ± 0.007	0.308 ± 0.002	0.325 ± 0.005
Hughes	0.358 ± 0.007	0.320 ± 0.004	0.323 ± 0.005	0.343 ± 0.004

**Table 5:** Five-fold cross-validation of the GLL values on SGD clusters

Datasets	POCS	**k-medoids **[10]	**ICM **[12]	**FCM **[13]
(a) *K *= 10				
alpha	-49 ± 3	-146 ± 8	-66 ± 2	-62 ± 2
cdc15	-47 ± 1	-148 ± 13	-67 ± 3	-61 ± 3
cdc28	-50 ± 2	-154 ± 14	-79 ± 3	-64 ± 3
elu	-52 ± 3	-152 ± 9	-69 ± 4	-61 ± 3
Hughes	-43 ± 3	-143 ± 11	-65 ± 4	-55 ± 3

(b) *K *= 15				
alpha	-42 ± 3	-171 ± 4	-69 ± 1	-64 ± 2
cdc15	-40 ± 1	-172 ± 4	-78 ± 4	-59 ± 3
cdc28	-43 ± 3	-169 ± 10	-79 ± 3	-64 ± 4
elu	-43 ± 1	-170 ± 13	-80 ± 3	-62 ± 3
Hughes	-39 ± 3	-167 ± 14	-62 ± 4	-53 ± 4

(c) *K *= 20				
alpha	-71 ± 3	-190 ± 8	-86 ± 2	-82 ± 2
cdc15	-74 ± 3	-194 ± 16	-89 ± 6	-79 ± 5
cdc28	-67 ± 3	-188 ± 14	-87 ± 2	-71 ± 2
elu	-82 ± 6	-197 ± 6	-89 ± 2	-88 ± 2
Hughes	-64 ± 4	-182 ± 11	-81 ± 5	-70 ± 4

(d) *K *= 25				
alpha	-64 ± 2	-216 ± 9	-91 ± 2	-78 ± 2
cdc15	-65 ± 4	-213 ± 17	-89 ± 6	-80 ± 6
cdc28	-62 ± 3	-216 ± 11	-89 ± 2	-77 ± 3
elu	-72 ± 3	-219 ± 14	-93 ± 2	-85 ± 4
Hughes	-63 ± 5	-204 ± 8	-84 ± 5	-67 ± 4

**Table 6:** Five-fold cross-validation of the NMI values on SGD clusters

Datasets	POCS	**k-medoids **[10]	**ICM **[12]	**FCM **[13]
(a) *K *= 10				
alpha	0.438 ± 0.008	0.383 ± 0.002	0.404 ± 0.002	0.408 ± 0.003
cdc15	0.462 ± 0.001	0.389 ± 0.004	0.422 ± 0.003	0.429 ± 0.004
cdc28	0.428 ± 0.005	0.387 ± 0.001	0.400 ± 0.002	0.411 ± 0.004
elu	0.432 ± 0.006	0.410 ± 0.004	0.411 ± 0.003	0.412 ± 0.004
Hughes	0.467 ± 0.004	0.414 ± 0.009	0.434 ± 0.003	0.439 ± 0.003

(b) *K *= 15				
alpha	0.533 ± 0.003	0.471 ± 0.006	0.507 ± 0.004	0.517 ± 0.004
cdc15	0.572 ± 0.002	0.507 ± 0.005	0.528 ± 0.005	0.540 ± 0.003
cdc28	0.552 ± 0.001	0.488 ± 0.004	0.524 ± 0.005	0.543 ± 0.003
elu	0.536 ± 0.008	0.466 ± 0.004	0.514 ± 0.003	0.525 ± 0.003
Hughes	0.566 ± 0.001	0.513 ± 0.007	0.549 ± 0.003	0.546 ± 0.005

(c) *K *= 20				
alpha	0.607 ± 0.003	0.551 ± 0.003	0.579 ± 0.004	0.583 ± 0.004
cdc15	0.613 ± 0.001	0.543 ± 0.005	0.580 ± 0.003	0.587 ± 0.004
cdc28	0.598 ± 0.002	0.551 ± 0.003	0.587 ± 0.004	0.586 ± 0.005
elu	0.593 ± 0.001	0.539 ± 0.006	0.567 ± 0.004	0.564 ± 0.003
Hughes	0.638 ± 0.004	0.576 ± 0.003	0.586 ± 0.005	0.591 ± 0.003

(d) *K *= 25				
alpha	0.649 ± 0.002	0.586 ± 0.004	0.636 ± 0.004	0.634 ± 0.006
cdc15	0.648 ± 0.006	0.594 ± 0.005	0.621 ± 0.004	0.620 ± 0.005
cdc28	0.661 ± 0.003	0.607 ± 0.005	0.630 ± 0.005	0.637 ± 0.006
elu	0.637 ± 0.004	0.607 ± 0.008	0.619 ± 0.006	0.621 ± 0.005
Hughes	0.667 ± 0.003	0.617 ± 0.009	0.637 ± 0.004	0.646 ± 0.005

**Table 7:** P-values of pairwise *t*-test of POCS and FCM

Number of clusters *K*	KEGG	SGD
10	1.60e-3	1.10e-3
15	1.29e-4	1.25e-2
20	1.30e-3	8.10e-3
25	2.80e-3	1.00e-3

To further confirm the effectiveness of POCS-based MGC method, we show two clustering examples. First, the gene YPR145W involves two KEGG pathways "Amino acid metabolism" and "Energy metabolism" in Table 1. All other MGC algorithms misclassify this gene into a single cluster, but our POCS algorithm successfully classify it into two clusters with probabilities 0.7 and 0.3. This example confirms the effectiveness of our method for identifying genes in multiple functions. Second, we examine the gene YJL052W involving two SGD pathways "glycolysis" and "gluconeogenesis" in Table 2. We compute the p-values between each gene function in GO and the cluster (alpha dataset when *K *= 10) containing the gene YJL052W using Gene Ontology Term Finder http://db.yeastgenome.org/cgi-bin/GO/goTermFinder.pl. We then rank the gene functions according to their p-values, and the top function is assigned to the gene cluster. We find that the top function is "glycolysis" with the p-value 3.12*e *- 41, which is consistent with one of SGD pathways in which YJL052W involves. This example further confirms that the discovered clusters indeed reflect the true biological functions in terms of pathways.

## Conclusion

This paper presents a novel MGC method within the generalized POCS framework, which successfully combines two constraints from different nature for gene clustering. In addition, we also propose the GLL to measure the soft clustering performance. Experimental results of five-fold cross-validation on different microarray datasets show that the POCS-based MGC method is competitive or superior to other state-of-the-art MGC methods based on KEGG and SGD reference gene clusters. In the future, we aim to incorporate more constraints such as DNA sequence features and gene network structures to improve gene clustering performance further. For example, the structural profiles of DNA sequences play important roles in key genetic processes such as transcription [39], replication [40], protein-DNA recognition [41], and tissue specificity [42]. We may use the similarity between structural profiles of DNA sequences as a new constraint for gene clustering. On the other hand, we may also develop more efficient supervised learning strategies to automatically determine the weights of simultaneous projections in Algorithm 1. For example, we may choose decision trees [43] or ensemble learning methods [44] to learn the weights of different constraints from training data, and apply these weights to clustering unknown genes for function prediction.

## Authors' contributions

JZ developed this methodology, carried out experiments and drafted the manuscript. ZSF and AWL provided useful comments on methodology and helped revise this manuscript. HY initiated the project and participated in project design and helped revise the manuscript. All authors read and approved the final manuscript.
